# Supplementary material for: The composite governance mechanisms and sustainable economic performance of Pakistan’s textile industry
Source: PLoS One. 2025 Feb 13;20(2):e0316388. doi: 10.1371/journal.pone.0316388 (PMC11825003; doi:10.1371/journal.pone.0316388)
Supplement: Attachment — Submitted filename: Letter to editor CGMI.doc [file pone.0316388.s002.doc]

**Date: 1-9-2024**

**Response Letter to the Editor**

The Editor,

PLOS ONE JOURNAL

Manuscript ID # PONE-D-24-17105

Dear Dr. Safdar Husain Tahir,

Thank you for allowing us to revise our manuscript (PONE-D-24-17105), entitled “The Composite Governance Mechanisms and Sustainable Economic Performance of Pakistan’s Textile Industry” for publication consideration in your journal. We very much appreciate the constructive criticism provided by the reviewers. Now we have made a sustained effort to address each issue raised by all three reviewers and to improve our manuscript accordingly.

In this revision, we have carefully addressed each point with the utmost diligence. Here are the important amendments we have made to the manuscript:

1. Please note, that in line with your data availability requirements, we have uploaded all data-supporting files for your kind perusal and record.
2. In addition, we have revised the abstract in a clearer and more informative manner. Please see page 1.
3. The introduction section is re-written and the justification for Pakistan's textile sector is highlighted. Please note that all changes are colored in red. Kindly see the page 4.
4. The study contributions are revised in line with the objectives of the study, this can be seen on page 6.
5. We have extensively revised the literature review sections. For the theoretical lens, please see page 7, 8, and 9. For the empirical relevance on CGMI-performance relationship, please see pages 12, 13 and 14.
6. We have rewritten the methodology section for more clarity and understanding. Sample selection criteria is re-stated, please see on pages 16, 17 and 18.
7. The description of Fixed effect, Random effect, and Hausman test are corrected, under section 3.4 “Panel Econometrics” on page 19, 23 and 25.
8. We have corrected the results and descriptions of all preliminary statistics. For Durbin Watson statistics please see page 21, and VIF Multicollinearity test, kindly see page 22. Endogeneity test results are described on pages 24-25.
9. We have re-calculated the panel regression analysis. A separate heading is added for each panel estimation model, please see pages 23-25.
10. Appropriate measures of variation and confidence intervals are added in Tables 5, 6, and 8.
11. Finally, the conclusion and discussion sections have been revised. Please refer to pages 26, 27, and 28.

Once again, we thank you to anonymous referees for their valuable comments and suggestions. We have approached these comments and suggestions with dedication and enthusiasm, as they have been a valuable learning experience.

We hope that you will see our manuscript in line with the PLOS ONE requirements.

Best regards,

Authors
